# *Ex Vivo* Models to Decipher the Molecular Mechanisms of Genetic Notch Cardiovascular Disorders

**DOI:** 10.1089/ten.tec.2020.0327

**Published:** 2021-03-15

**Authors:** Tommaso Ristori, Marika Sjöqvist, Cecilia M. Sahlgren

**Affiliations:** ^1^ Department of Biomedical Engineering, Technical University of Eindhoven, Eindhoven, The Netherlands.; ^2^ Institute for Complex Molecular Systems, Eindhoven University of Technology, Eindhoven, The Netherlands.; ^3^ Department of Biomedical Engineering, Boston University, Boston, Massachusetts, USA.; ^4^ Faculty of Science and Engineering, Biosciences, Åbo Akademi University, Turku, Finland.; ^5^ Turku Bioscience Centre, Åbo Akademi University and University of Turku, Turku, Finland.

**Keywords:** *ex vivo*, *in vitro*, *in silico*, Notch, cardiovascular

## Abstract

Notch is an evolutionary, conserved, cell–cell signaling pathway that is central to several biological processes, from tissue morphogenesis to homeostasis. It is therefore not surprising that several genetic mutations of Notch components cause inherited human diseases, especially cardiovascular disorders. Despite numerous efforts, current *in vivo* models are still insufficient to unravel the underlying mechanisms of these pathologies, hindering the development of utmost needed medical therapies. In this perspective review, we discuss the limitations of current murine models and outline how the combination of microphysiological systems (MPSs) and targeted computational models can lead to breakthroughs in this field. In particular, while MPSs enable the experimentation on human cells in controlled and physiological environments, *in silico* models can provide a versatile tool to translate the *in vitro* findings to the more complex *in vivo* setting. As a showcase example, we focus on Notch-related cardiovascular diseases, such as Alagille syndrome, Adams–Oliver syndrome, and cerebral autosomal dominant arteriopathy with subcortical infarcts and leukoencephalopathy (CADASIL).

**Impact statement:**

In this review, a comprehensive overview of the limitations of current *in vivo* models of genetic Notch cardiovascular diseases is provided, followed by a discussion over the potential of microphysiological systems and computational models in overcoming these limitations and in potentiating drug testing and modeling of these pathologies.

## Introduction

Notch is a signaling pathway that relies on direct cell–cell interaction for its activation. Notch ligands (Dll1, Dll4, Jag1, and Jag2) presented at the surface of a cell interact with membrane-bound Notch receptors (Notch1–4) of another adjoining cell. The ligand–receptor interaction results in proteolytic processing of the Notch receptor, releasing the active signaling unit of the receptor, the Notch intracellular domain (NICD). Upon its release into the cytoplasm, the NICD translocates into the nucleus where it binds RBPJk, thereby inducing transcription of target genes and activation of transcriptional programs. This contributes to the determination of cell fate and regulation of tissue development and homeostasis.

In the cardiovascular system, Notch controls heart morphogenesis as well as blood vessel formation^1–3^ and homeostasis.^4,5^ Concurrently, mutations in different Notch ligands or receptors can cause several pathologies such as congenital heart defects (CHDs) and diseases such as Alagille syndrome (ALGS), Adams–Oliver syndrome (AOS), calcific aortic valve disease (AoVD), and cerebral autosomal dominant arteriopathy with subcortical infarcts and leukoencephalopathy (CADASIL).^3,6^

**FIG. 1.**
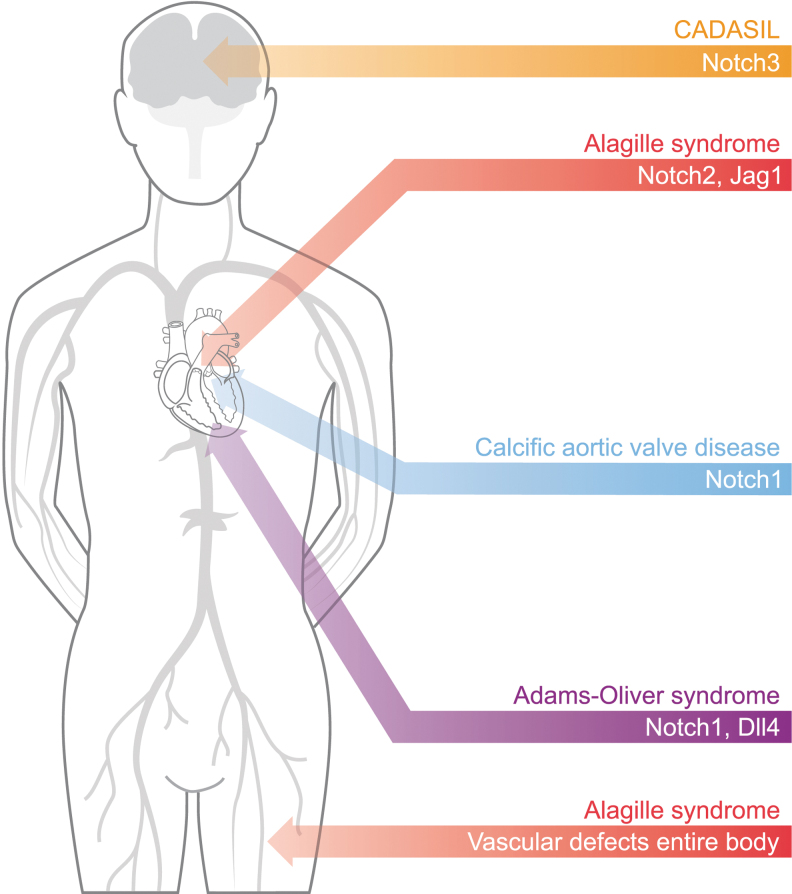
Mutation of Notch signaling components can cause different congenital pathologies, especially within the cardiovascular system. Notch3 mutations lead to CADASIL, which mainly affects the brain vasculature. Notch1 mutations cause heart diseases as they are associated with both calcific AoVD and AOS, with the latter also caused by Dll4 mutations. Finally, mutations of Jag1 or Notch2 can lead to ALGS that presents vascular defects throughout the body and heart defects. ALGS, Alagille syndrome; AOS, Adams–Oliver syndrome; AoVD, aortic valve disease; CADASIL, cerebral autosomal dominant arteriopathy with subcortical infarcts and leukoencephalopathy.

ALGS, AOS, and CADASIL are autosomal dominant diseases that display cardiovascular symptoms.^3,6^ Each of these diseases is associated with mutations in genes coding for different Notch components (Fig. 1). ALGS is a multisystemic pediatric disease caused by mutations in *JAG1* or *NOTCH2*. The vascular complications include spontaneous bleeding, aneurysms, moyamoya syndrome, renal stenosis, and dilation of carotid arteries,^7,8^ as well as different CHDs such as tetralogy of Fallot, pulmonary stenosis, ventricular septal defects, and patent ductus arteriosus.^9^

Mutations in *NOTCH1* are associated with aberrant morphogenesis of the aortic valves, occurrence of AoVD,^10^ and AOS.^11^ This latter syndrome is also caused by mutations in *DLL4*,^12^ *RBPJk*,^13^ and other Notch-modifying genes. Despite AOS mainly manifesting through terminal transverse limb defects and scalp aplasia cutis, several AOS patients also display CHDs.^14,15^ CADASIL is a vascular dementia syndrome caused by mutations in *NOTCH3*, resulting in degeneration of vascular smooth muscle cells (VSMCs) and white matter, together with ischemic strokes.^16^ Unfortunately, current treatments cannot cure these diseases and only aim at mitigating the symptoms. To overcome this limitation and develop new regenerative therapeutic strategies, further understanding of the underlying molecular mechanisms is required.

Despite several efforts, current *in vivo* disease models fall short of replicating the actual human disease characteristics, which suggests that fundamental differences between species are present in this context. At the same time, the inherent complexity of *in vivo* models complicates the identification of the exact molecular mechanisms involved, thereby hindering the development of new therapies. In this perspective review, after summarizing current *in vivo* models of Notch-related cardiovascular diseases, we propose that their limitations could be overcome by combining engineered *in vitro* models, providing a controlled environment to investigate specific aspects of the pathologies, together with *in silico* models, providing a digital twin for the *in vivo* setting.

## *In Vivo* Mouse Models for Notch-Associated Diseases

*In vivo* animal models are generally the most reliable in mimicking the complexity of human organisms. As such, they have been widely adopted to screen for the effects of Notch mutations. However, although ALGS, AOS, AoVD, and CADASIL are caused by autosomal dominant mutations, cardiovascular phenotypes are not obvious in mice presenting targeted disruption of one allele in the associated Notch genes,^17–19^ except for *Dll4* in AOS.^20–22^ This limitation suggests that murine models are not fully reliable in this case as mice might have differences or compensatory mechanisms masking the effects of Notch mutations.

For example, mice most likely exhibit a different hemodynamic environment compared to humans, which can influence Notch responses to mechanics.^23^ Furthermore, mouse models have a short life span and different hallmarks of aging, which affect the possibility to mimic the temporal features of Notch-related diseases, including the fact that the first symptoms of CADASIL start to become evident in young adults^16^ despite the Notch-related mutation being present since birth. Nevertheless, a few animal models replicate some details of cardiovascular Notch-related diseases and thus provide valuable information on the disease etiology.

Mice carrying an array of different Notch mutations have been proposed as ALGS models.^24–29^ While mice heterozygous for either *Jag1* or *Notch2* cannot mimic the disease,^17,19^ double heterozygous mice (*Jag1*^dDSL/+^*Notch2*^del1/+^) can recapitulate several ALGS symptoms, including cardiac defects.^24^ However, ALGS is known to arise from single mutations in *JAG1* and, more rarely, *NOTCH2*. Recently, Andersson *et al.*^27^ have described a novel mouse (*Jag1*^Ndr/Ndr^) with a phenotype mimicking ALGS in humans. The proposed mutation renders JAG1 hypomorphic, both inhibiting signaling through NOTCH1 and hindering activation of NOTCH2 and NOTCH3.^27^ This new model suggests that ALGS may result from Notch mutations causing partial loss of function, leading to reduced Notch activity. Overall, not only do these findings highlight the importance of understanding the specific roles of different Notch components in human physiology but they also point to the necessity of further analyzing Notch dose sensitivity.

Several mouse models of CADASIL, expressing mutant variants of NOTCH3 with altered numbers of cysteines, partially recapitulate the CADASIL phenotype. These mice exhibit the characteristic deposition of granular osmiophilic material and accumulation of NOTCH3, but no evident brain lesions^30,31^ (for a review, see^32^). Differently, introduction and overexpression of NOTCH3^R169C^ into mice in a heterozygous state successfully created a phenotype also displaying white matter degeneration.^33^ This model has recently led to new findings suggesting that CADASIL is caused by Notch3 gain-of-function mutations that cause increased NAPDH oxidase 5 activation and consequential endoplasmic reticulum stress.^34^ Similar hypermorphic associations have been made with mutant Notch3 and NF-κB signaling, aggravating vascular remodeling and inflammation.^35^ However, this NOTCH3^R169C^ model required excessive overexpression of the mutant protein, above endogenous levels,^33^ and whether CADASIL is caused by loss- or gain-of-function mutations is still strongly debated.^32,36,37^ Future studies should aim at clarifying this point, in addition to identifying the mechanisms causing CADASIL symptoms to start appearing in young adults and mainly in the brain region. Characterizing the temporal progression and localization of the disease might lead to development of strategies for early diagnosis and treatments.

Mice heterozygous for either *Notch1* or *RBPJk* can mimic the human phenotype of AoVD to some extent. In particular, *Notch1*^+/−^ mice show enhanced valve calcification and activation of alkaline phosphatase.^38^ *RBPJk*^+/−^ mice display greater cardiac impairment, including thickened, calcified valve leaflets, macrophage infiltration, and collagen deposition, as well as activation of procalcific and profibrotic signaling pathways.^39^ Moreover, *RBPJk*^+/−^ mice also have upregulated levels of Runx2,^39^ a transcription factor promoting osteoblast fate and heavily upregulated in human AoVD.^40^ The seemingly important role of RBPJk^39^ suggests that AoVD is a result of generally disrupted Notch transcriptional activation, which, given the results with the Notch1 mice,^38^ mainly occurs through Notch1, although other receptors must be involved. Unfortunately, *Notch1*^+/−^, *RBPJk*^+/−^, and *Dll4*^+/−^ mice are, however, not representative models of AOS as they do not display the terminal transverse limb defects and scalp aplasia cutis characteristic of AOS. It has been proposed that vascular defects would be the main drivers of AOS,^41,42^ and mice with induced inhibition of Notch activity display loss of VSMC coverage and hemorrhage in different regions of the developing brain and limbs.^41^ In conclusion, animal models of Notch-associated cardiovascular diseases are currently not completely satisfactory; therefore, alternative and complementary strategies are necessary.

## *In Vitro* Microphysiological Systems

Failure to meet the expectations of drug development and drug screening incentives has driven the development of microphysiological systems (MPSs) or organs-on-chips that recapitulate the function of human organs.^43^ MPSs can also aid in elucidating molecular mechanisms underlying human pathologies and the identification of therapeutic targets. As described above, this can be difficult in animal models, which may not reflect human pathophysiology and where dynamic molecular signaling events and complex cell–microenvironment interactions are difficult to capture. This is especially true for the cardiovascular system, where changes in hemodynamics can critically affect Notch signaling.^23^ Furthermore, many of the Notch-associated cardiovascular disorders listed above are rare disorders.^44^

Identifying molecular therapeutic targets and treating rare disease patients are huge challenges due to the limited amount of data available and the lack of relevant model systems. Fewer than around 5% of around 7000 currently identified rare diseases have effective drug therapies,^45^ and modeling rare disease has high potential to overcome this issue. Complex and rare diseases can be modeled in MPSs^46^ using cells from patient donors, either from primary or induced stem cell sources, or using genetic tools to induce a disease phenotype that can be studied *in vitro* against an isogenic background.

**FIG. 2.**
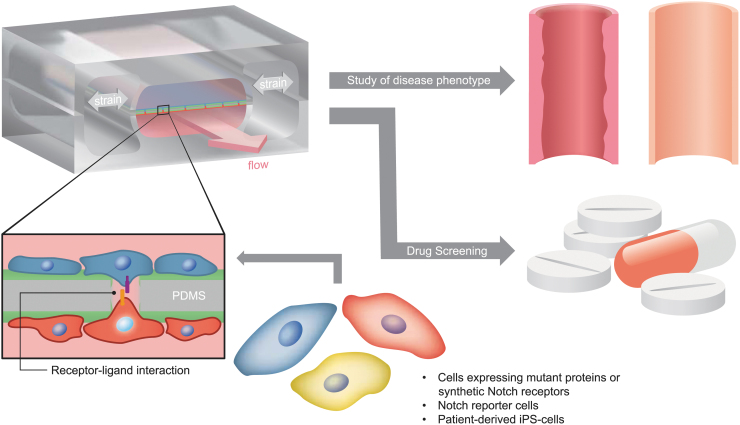
Microphysiological systems mimicking specific features of the cellular environment can enable the study of diseases caused by Notch mutations. For example, patient-derived or other human cells can be inserted in microfluidic devices mimicking the layer separation between endothelial cells and VSMCs, which is evident in blood vessels not only in terms of location but also stimuli (shear stress and cyclic stretch for endothelial cells and only cyclic stretch for VSMCs). These devices provide platforms where Notch interactions can be efficiently quantified, thereby enabling the controlled investigation of disease phenotypes and drug screening with donors' cells (the scheme of the microfluidic device is adapted from Ref. van Engeland et al.^61^). VSMC, vascular smooth muscle cell.

The Hutchinson–Gilford progeria syndrome (HGPS) is a rare genetic condition that is caused by a mutation in the Lamin A gene. Recently, a tissue-engineered blood vessel model of HGPS using induced pluripotent stem cell (iPSC)-derived endothelial cells identified the role of the endothelium in HGPS.^47^ Other rare diseases that have been modeled using iPSCs and organ-on-chip technologies include hereditary hemorrhagic telangiectasia, Rett syndrome, Alpers–Huttenlocher syndrome (as reviewed by Low and Tagle^46^), and Barth syndrome, where a heart-on-chip technology provided new insights into the pathogenesis.^48^ For all these reasons, we expect that the MPS will be a game changer also in the investigation of genetic Notch disorders as it could be adopted to study disease-mimicking cells and test drugs under physiologically relevant conditions (Fig. 2).

As described in the sections above, most of the genetic Notch cardiovascular disorders seem to arise from defects in blood vessels, which are in turn caused by incorrect temporal changes in Notch dosage,^27^ mediated by the interactions between multiple Notch ligands and receptors,^27,38,39^ each having a specific role. To elucidate these mechanisms, therefore, techniques able to engineer cells expressing different (ratios of) Notch ligands and receptors in a controlled manner should be adopted, and MPSs should enable long-term culture of cells in environments mimicking the structure, components, and stimuli of blood vessels.

Possible disease-mimicking cells might be obtained through synthetic biology and cellular engineering, which have already helped in elucidating Notch signaling networks and the impact of receptor–ligand specificity on signaling dynamics.^49,50^ Notch signaling dynamics is important for tissue development and morphogenesis,^49,51,52^ and Notch signaling dose and specific receptor–ligand interactions affect cell fate^53,54^ and physiology.^20^ Advances in synthetic biology, stem cell, and organoid technologies, as well as gene editing, combined with advanced microengineering of humanized model systems, will drive cutting-edge translational research.

Within vascular biology, three-dimensional (3D), *ex vivo* vascular networks have been useful to study the impact of Notch signaling dynamics on tissue morphogenesis and malformation,^55^ but these systems lack the impact of mechanics. Coupling dynamic molecular activity and mechanics to tissue pathology, to understand the interrelationship between biochemical regulation and mechanical impact on cardiovascular disease, requires real-time monitoring of molecular and cellular activities in humanized physiological systems and control over hemodynamic parameters. *In vitro* models with simplified mechanical characteristics or reduced tissue complexity, *ex vivo* microfluidic channels, and cellular strain platforms have all been used to study the mechanosensitivity of Notch in vascular cells.^4,56–59^ The complexity of these systems should be gradually increased to more closely mimic the physiological environment.

The hemodynamic environment of blood vessels consists of both shear stress (due to blood flow) and circumferential stretch (due to blood pressure). The tissue is layered, multicellular, and 3D. While simple microfluidic or strain devices can be employed to assess the impact of mechanics on Notch, more complex models and tissue-engineered constructs are a step closer toward mimicking the complexity *in vivo*. Perfused, self-organized vascular systems in 3D gels have helped in elucidating novel mechanisms of Notch in vascular barrier functions.^60^

We recently developed a vessel wall on a chip, mimicking the physiological cell composition, organization, and hemodynamic environment of the arterial vessel wall, to study the impact of simultaneous shear stress and strain on endothelial-VSMC signaling in tissue remodeling.^61^ A bioreactor that independently combined shear stress and stretch was developed to study the development of vascular grafts,^62^ and another bioreactor with a controlled, pressurized tissue system was developed to study the long-term effect of constant strain versus constant stress on tissue growth.^63^

In sum, several bioreactors and MPSs have already been developed and hold great promise in mimicking several aspects of genetic Notch cardiovascular disorders. The modeling of disease states on MPSs opens new avenues for understanding molecular mechanisms of pathologies and potential treatments of Notch-linked vascular disease. These models will provide insight into complexity, dose sensitivity, and receptor–ligand specificity of Notch signaling in cardiovascular tissue and especially in relation to changes in tissue mechanics and hemodynamics.

## *In Silico* Models Bridging *In Vitro* and *In Vivo* Models

Although MPSs enable the investigation of human diseases with human donor cells and the investigation of isolated stimuli acting on them, *in vitro* findings remain challenging to translate to *in vivo* settings, where several biological processes interact, creating feedback loops with counterintuitive results. This conceptual gap between *in vitro* and *in vivo* models may be bridged by *in silico* models, providing digital twins of *in vivo* conditions and analytic tools for understanding the systemic consequences of single biological mechanisms.

*In silico* models have a wide range of applications within the investigation of vascular diseases and associated treatments. For example, computational fluid dynamics models can potentiate the optimization of *in vitro* systems^64–66^ and analysis of the effects of vascular network geometry on hemodynamics.^67–69^ Not only can these models compute the shear stress experienced by endothelial cells and other cell types *in vitro*^70–72^ or in human pathologic conditions^73^ but they can also be adopted to optimize surgical strategies for Notch-related diseases such as ALGS.^74–76^

Similarly, computational models are widely adopted to compute the local strain experienced by cardiovascular cells resulting from blood pressure-induced tissue deformation,^77–79^ both for idealized and patient-specific geometries.^80,81^ Coupled with this, an increasing number of modeling approaches include the possibility to simulate cell behavior in response to chemo-mechanical cues within cardiovascular tissues.^82^ Given the growing evidence of Notch mechanosensitivity, the capability of computational models to analyze cellular mechanical stimuli will be increasingly crucial for the study of Notch-related diseases. To this aim, these biomechanical models have to be coupled with *in silico* Notch models.

Due to the importance of Notch in numerous contexts, many *in silico* models of this signaling pathway have been proposed,^83^ usually to investigate the effects of different Notch ligand–receptor relationships on the resulting cellular phenotypic patterns.^84–89^ For example, modeling studies have uncovered that spatial patterns of cells with two alternating phenotypes can arise both from Notch lateral inhibition,^84^ defined as downregulation of Notch ligands in receiving cells after Notch activation, and from cis-inhibition,^85^ that is, the mutual exclusion of Notch receptor–ligand pairs within a single cell without Notch activation. Similarly, simulations have been crucial to understand the effects of filopodia,^90,91^ cell area and size,^88^ mechanics,^4,92,93^ and cell connectivity^89^ on Notch signaling dynamics and consequential tissue patterning. To our knowledge, there are currently no computational studies simulating Notch signaling in genetic Notch cardiovascular disorders. Nevertheless, we envision that combining and extending the features of the existing Notch *in silico* models will provide great versatility and opportunities for investigation of Notch-related diseases and for acceleration of targeted drug development and approval as it is also suggested and currently occurs in other fields.^94,95^

**FIG. 3.**
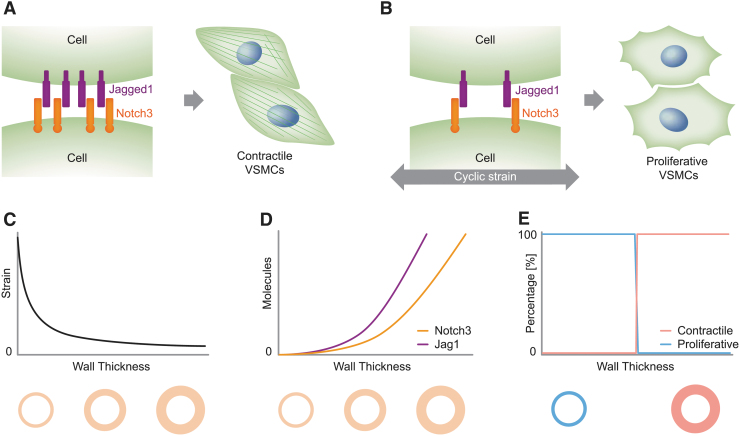
*In silico* models can translate in vitro findings to the *in vivo* setting. For example, *in vitro* studies have shown that the phenotype of VSMCs is strongly influenced by Notch signaling and mechanics: **(A)** high Notch activation corresponds to contractile VSMCs; **(B)** cyclic strain downregulates the expression of Notch3 and Jag1, and low levels of Notch correspond to proliferative VSMCs. By accounting for the fact that **(C)** the strain in arteries decreases with wall thickness and, consequently, **(D)** Notch3 and Jag1 expression levels increase with arterial thickness, **(E)** a recent computational model^4^ predicted that Notch mechanosensitivity can induce a phenotypic switch of VSMCs from proliferative to contractile for a specific wall thickness, thereby suggesting a mechanism that regulates the arterial homeostatic thickness (**A** and **B** are adapted from Ref. Loerakker and Ristori^82^).

For example, our group has recently proposed a computational model for Notch signaling in blood vessels, which might have several applications in investigating CADASIL and ALGS.^4^ By accounting for Notch3-Jag1 signaling and their sensitivity to cyclic strain, this model identified Notch mechanosensitivity as a possible key factor of blood vessel homeostasis^4^ (see Fig. 3 for a scheme). The predictive potential of this model is supported by further experiments from our group.^93^ Future studies could adopt and extend this model to unravel the underlying mechanisms of CADASIL.

In fact, the proposed Notch circuit model could be extended to investigate the possible effects that CADASIL-induced accumulation of the Notch3 extracellular domain^96^ has on Notch signaling and vascular homeostasis. Moreover, if coupled with simulations of blood vessel mechanics during aging, the model might shed light on the effects that age-related modifications have on Notch3-Jag1 signaling and, in turn, on the appearance of CADASIL symptoms in young adults.^16^ Finally, the Notch circuit model could be extended to include other Notch receptors, with different effects and behaviors, thereby enabling the investigation of ALGS dose sensitivity and Notch receptor specificity, which was discussed in the section on *in vivo* mouse models.

Conditional for their success, computational models need to be tightly coupled to experiments, which are strictly necessary for the model-informed development and validation. In fact, it is increasingly clear that each Notch ligand and receptor has its own specific role and regulation,^49,50,54^ which depend on the environment and, most likely, cell type. For example, Notch activation by Dll1 and Dll4 has different effects.^49^ Similarly, the activity of the enzymes, manic and lunatic fringe, is known to reduce Jag1-Notch2 binding^97^ and, on the opposite, increase Jag1-Notch1 binding events.^98,99^ On the one hand, accounting for these types of distinctions is not important for the quality of computational studies analyzing Notch signaling in general; on the other hand, it is crucial for the reliability of simulations of more specific biological phenomena, such as Notch-related diseases. Similarly, Notch3 activity might have location-dependent roles as it has opposite effects in the progression of arterial and pulmonary hypertension.^100,101^ Therefore, fully reliable *in silico* models can be attained only by informing their development with targeted experiments that analyze the specific cell type and specific Notch proteins. Future efforts in this field should thus aim at potentiating the interplay between experiments and simulations by establishing more rigorous methods for the calibration and validation of *in silico* models based on cell experiments.

## Conclusion and Outlook

Unfortunately, current animal models of Notch-associated cardiovascular diseases are still not fully satisfactory. Although (in some cases) they have helped in understanding whether these pathologies result from loss- or gain-of-function Notch mutations, this information is still not enough for development of drugs targeting the disease instead of the symptoms. To this aim, fully understanding the molecular pathways involved is necessary. Achieving such understanding solely with animal models is challenging, if not impossible, not only because of their incapability to fully recapitulate Notch-related diseases but also because of their complexity. A further challenge stems from potential species differences. In general, the insufficient contribution of animal models is also highlighted by the disproportionate number of drugs that positively pass animal trials, but fail to reach the clinical setting due to negative results in humans.^102^ Alternative and complementary strategies are therefore necessary.

**FIG. 4.**
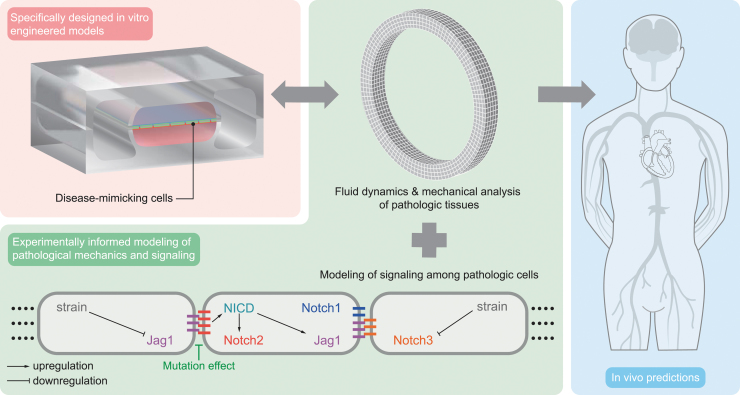
Example of workflow. Specifically designed *in vitro* engineered models are adopted to mimic the *in vivo* environment and test disease-mimicking cells. The *in vitro* experiments inform the development of computational models of tissue mechanics and Notch signaling as perturbed by the genetic mutation under investigation. The computational model predictions can be validated with further *in vitro* experiments, completing the cycle of model information and validation. Ideally, this process should lead to increased model credibility and reliable *in vivo* predictions (the scheme of the microfluidic device is adapted from Ref. van Engeland et al.^61^).

We expect that the combination between MPSs and computational models will significantly aid our understanding of Notch signaling mechanisms in cardiovascular disorders and will potentiate the development of targeted treatments (Fig. 4). MPSs provide controlled environments mimicking isolated features of human physiology, where patient-derived or other human cells can be directly cultured and relatively easily tested and imaged. Computational models can integrate findings from different experiments and predict their consequences *in vivo*, where multiple phenomena interact over a long time period. Therefore, these two approaches complement each other and could lead to breakthroughs in the field of Notch-related cardiovascular diseases.

Future MPSs should aim at more closely mimicking the complexity of human tissues and conditions while keeping their advantages in terms of control. Further complexity and physiological relevance may be achieved by including more features of the systemic environment, such as gradients of nutrients, gasses, cytokines, hormones, circulating cells, and other relevant cell types, and by tuning the composition and organization of the extracellular matrix. Ideally, this should be performed following a modular design so that complexity can be increased or decreased depending on the research question. Such an approach should also be followed by computational models to increase their versatility and enable their application in different contexts.

Importantly, given the long timescale features of Notch-related diseases and tissue morphogenesis, growth, and remodeling more in general, both MPSs and computational models should reach the potential to recapitulate long-term organ function, enabling long-term cell culture on the one hand and long-term simulations with contained computational costs on the other hand. Reaching these goals remains challenging and requires engineering skills that are not yet widely accessible. To fill this gap, we need to develop new tools and a new framework where expertise in cell biology, molecular biomedicine, engineering, and computational biology is combined. To facilitate communication and the exchange of information and tools between these different disciplines, common hybrid international conferences should be organized and strategies should be optimized to increase the accessibility of tissue chip technology and computational models. For this latter aim, these technologies should be accurately validated and commercialized for community-wide access.

In conclusion, the coupling and further development of MPSs and computational models may accelerate the investigation of genetic Notch cardiovascular disorders and could provide new, personalized medicine approaches as drugs may be tested directly on patient-derived cells within MPSs mimicking the human physiology and disease. The potential of this coupling is currently largely unexplored in this field and therefore, despite the challenges ahead, we envision that the interplay between these two approaches will lead to accelerated drug discovery and an increased approval rate, in addition to facilitating the transition from care, where only symptoms are treated, to cure, where disease causes are targeted.
